# Predictors of glycosylated haemoglobin A1C trend among type 2 diabetes patients in a multi-ethnic country

**DOI:** 10.1038/s41598-021-86277-0

**Published:** 2021-03-24

**Authors:** Kim Sui Wan, Noran Naqiah Hairi, Feisul Idzwan Mustapha, Khalijah Mohd Yusof, Zainudin Mohd Ali, Foong Ming Moy

**Affiliations:** 1 Centre for Epidemiology and Evidence-Based Practice, Department of Social and Preventive Medicine, Faculty of Medicine, University of Malaya, Jalan Universiti, 50603 Kuala Lumpur, Malaysia; 2 Disease Control Division, Ministry of Health, Level 3 Block E10, Kompleks E, Pusat Pentadbiran Kerajaan Persekutuan, 62590 Putrajaya, Malaysia; 3 State Health Department of Johor, Jalan Persiaran Permai, 81200 Johor Bahru, Johor Malaysia; 4 State Health Department of Negeri Sembilan, Jalan Rasah, 70300 Seremban, Negeri Sembilan Malaysia

**Keywords:** Endocrine system and metabolic diseases, Endocrinology, Risk factors, Public health

## Abstract

Good control of glycosylated haemoglobin A1C in diabetes patients prevents cardiovascular complications. We aim to describe the A1C trend and determine the predictors of the trend among type 2 diabetes patients in Malaysia. Longitudinal data in the National Diabetes Registry from 2013 to 2017 were analysed using linear mixed-effects modelling. Among 17,592 patients, 56.3% were females, 64.9% Malays, and the baseline mean age was 59.1 years. The U-shaped A1C trend changed marginally from 7.89% in 2013 to 8.07% in 2017. The A1C excess of 1.07% as reported in 2017 represented about 22% higher risk of diabetes-related death, myocardial infarction, and stroke, which are potentially preventable. The predictors for higher baseline A1C were non-Chinese ethnicity, younger age groups, longer diabetes duration, patients on insulin treatment, polypharmacy use, patients without hypertension, and patients who were not on antihypertensive agents. Younger age groups predicted a linear increase in the A1C trend, whereas patients on insulin treatment predicted a linear decrease in the A1C trend. Specifically, the younger adults and patients of Indian and Malay ethnicities had the poorest A1C trends. Targeted interventions should be directed at these high-risk groups to improve their A1C control.

## Introduction

Cardiovascular disease (CVD) is the number one cause of death globally and in Malaysia^1,2^. Patients with diabetes have a two-fold excess risk in developing CVD, independently of other risk factors^3^. CVD is responsible for more than half of the deaths among patients with type 2 diabetes (T2D)^4^. The prevalence of diabetes among Malaysian adults has increased from 11.2% in 2011 to 18.3% in 2019^5^.

Study findings from the United Kingdom Prospective Diabetes Study (UKPDS) signify the importance of glycosylated haemoglobin A1C control in preventing microvascular and macrovascular complications, including deaths^6^. The long-term follow-up study demonstrated that early tight glycaemic control had sustained benefits. The reduction in microvascular and macrovascular events persisted despite the loss of glycaemic differences between the intensive-therapy group and the conventional-therapy group, an observation known as the legacy effect^7^.

Monitoring of the A1C trend is crucial as it reflects diabetes care quality in healthcare facilities^8^. About 7.4% of cardiovascular events (myocardial infarction, acute coronary syndrome, stroke, or heart failure) in diabetes patients without CVD were associated with inadequate A1C control. These outcomes were preventable if additional attention was given to control glycemia^9^.

With the advent of new antidiabetic treatment and advances in diabetes care programmes, it is reasonable to expect a substantial improvement in CVD risk factors control^10^. However, a review reported that diabetes patients in Singapore had marginal glycaemic improvements over 20 years^10^. On the other hand, a 5-year longitudinal study in Hong Kong reported a considerable improvement in mean A1C^11^.

The Malaysian National Diabetes Registry reported a marginal improvement of mean A1C over the last 10 years^12,13^. However, this result was based on repeated cross-sectional analysis which did not reflect intra-individual changes over time, and the predictors of A1C trend were also not investigated. In the emerging practice of precision public health, it is essential to predict and understand public health risks and tailor interventions for at-risk groups to improve the overall population health^14^. Delivering the right intervention at the right time and to the right population is the goal. Hence, this study aims to describe the A1C trend and determine the predictors of the trend among T2D patients in Malaysia.

## Methods

This was a 5-year retrospective open cohort study from 2013 to 2017. The study population was T2D patients treated in all public health clinics in Negeri Sembilan, Malaysia. About 70% of diabetes patients in Malaysia received diabetes treatment from public health clinics^5^. Negeri Sembilan is located to the south of the capital city Kuala Lumpur. There were several reasons for choosing the state as the study location. Negeri Sembilan has the highest prevalence of diabetes in Malaysia; at 33.2%, its prevalence is much higher than the national average (18.3%)^5^. The composition of the general population in Negeri Sembilan by age, sex, and ethnicity are comparable to the national composition^15^. Similarly, the degree of urbanisation in Negeri Sembilan (75.9%) closely approximated the national average (76.2%)^16^. In addition, the diabetes data quality was potentially better in Negeri Sembilan as the state contributed 20% of the audit data in the National Diabetes Registry^12^. The state has consistently conducted audits on the registry database. Moreover, the demographic characteristics of T2D patients from Negeri Sembilan are comparable to the national average^12^.

The inclusion criteria of our study participants were patients with T2D, aged 18 years and above, and had at least two clinical audits between 2013 and 2017. Patients entered this open cohort from 2013 to 2016 and exited the cohort between 2015 to 2017. Patients with pre-existing CVD were excluded.

The cohort data were extracted from the National Diabetes Registry, a surveillance database to monitor diabetes control and clinical outcomes among patients receiving treatments from public healthcare facilities^12^. Annually, a subset of T2D patients was randomly sampled for clinical audits. All patients had the same probability of being selected, independent of whether they had been audited before^12^. T2D patients would usually have several visits to diabetes clinics each year, and their last observed clinical information was used in the audits to represent their whole year performance.

The primary outcome of this study was the A1C trend from 2013 to 2017. The A1C goal was set at < 7.0% following the clinical recommendation by the International Diabetes Federation^17^. As patients were randomly and independently sampled for clinical audits each year, there could be missing A1C data between these 2 audited years. The pattern of missingness was missing completely at random (MCAR) and therefore did not bias the observed data^18^.

The baseline characteristics such as demographic, smoking, comorbidities, complications, and treatment profiles were captured when patients entered the study. The demographic factors were age, sex, and ethnicity. The age categories were 18–49, 50–59, and ≥ 60 years. The ≤ 49 years category corresponded to the upper limit of the female reproductive age range according to the Department of Statistics, Malaysia^19^. Older adults were patients aged ≥ 60 years based on the United Nations’ definition^20^. The ethnic groups were Malays, Chinese, Indians, and other ethnicities. Smoking status was yes/no to ever smoking. The comorbidities were overweight and obesity following the World Health Organization body mass index (BMI) classification^21^, dyslipidaemia as diagnosed clinically or using a lipid-lowering agent, and hypertension as diagnosed clinically or using an antihypertensive agent. Diabetes complications, namely retinopathy, nephropathy, and foot complication, were based on clinical diagnoses. The treatment profiles included diabetes treatment modality, the use of antihypertensive, lipid-lowering, and antiplatelet agents. Other variables were duration of diabetes and polypharmacy, which was the use of five or more types of agents^22^.

### Statistical analysis

For normally distributed data, variables were presented in mean ± standard deviation (SD), while median and inter-quartile range (IQR) were presented for skewed data. Both frequency and percentages were reported for categorical variables. The 95% confidence intervals were also presented for the proportion of patients achieving the A1C goal.

Linear mixed-effects modelling (LMM) method was used to determine the A1C trends. This method was selected over general linear models (GLM) (e.g., repeated measures ANOVA) for the following reasons^23^: (a) unequally spaced and unbalanced dataset due to open cohort design, random sampling for clinical audit, and missing data increased type one error in the GLM method. However, this was accounted for in the LMM method; (b) missing data in the GLM method were list-wise deleted while in the LMM method, only missing data points were dropped from the analysis and all the remaining data points were included in the analysis; (c) the assumption of independence of observations was violated in the GLM method. Repeated measurements in the same patients were usually correlated and not independent; (d) the LMM method could study within- and between-patient differences, whereas the GLM method only focused on group differences, and (e) the effects of predictors could be more flexibly added using the LMM method.

Individual growth curves using two-level hierarchical models were created^23^. The level one model nested time within the individuals and showed the within-patient A1C trend from 2013 to 2017. The trend had two components: the intercepts estimated the initial mean A1C in 2013 while the slopes estimated the rates of A1C change. As there was a maximum of five observations for each patient, higher-order growth parameters (quadratic time^2^ and cubic time^3^) were also tested if the basic linear (time) growth model was statistically significant; this yielded a better overall picture of the trend. The level two model determined whether the differences between patients varied systematically. Predictors were first added individually into the growth model (univariate analysis). Their interactions with the growth parameters were also evaluated by adding the interaction term of predictor × time. The predictors, intercepts, and growth parameters were treated as fixed effects, whereas the intercepts and growth parameters were allowed to vary across individuals as random effects^23^.

A multivariate linear mixed-effects model was formed by including statistically and clinically significant predictors (and the predictor’s interaction with time). Model fit was examined using the Akaike information criterion, Bayesian information criterion, and -2 log-likelihood; information criteria were in smaller-is-better form. We used the maximum likelihood estimation as we focused on both fixed and random effects^24^. Different error covariance structures (unstructured, first-order autoregressive, and compound symmetric) were examined to find the best structure that fitted the data, which was important in an unequally spaced and unbalanced dataset^23^. The A1C estimates, *P*-values, and 95% confidence intervals were reported.

In post hoc analysis, the relationship between age and diabetes duration was tested using Spearman’s rank-order correlation. The characteristics of several ethnicities and age groups were compared to explore potential reasons for the differences in the study findings. Parametric one-way ANOVA and non-parametric Kruskal–Wallis tests were carried out for continuous variables. For categorical variables, Pearson chi-square tests were conducted. The tests were two-tailed, and a *P*-value of less than 0.05 was considered statistically significant. All analyses were carried out using the IBM SPSS Statistical Software Version 23.

Our study utilised secondary data with personal information and patient identifiers removed. All cases were anonymised and there was no interaction with any patients. Written informed consent was not required following local legislation and national guidelines. Waiver of informed consents was approved by the Medical Review and Ethics Committee (MREC), Ministry of Health, Malaysia. This study was approved and registered with the National Medical Research Registry of Malaysia (NMRR-18-2731-44032-IIR). All methods of this study were conducted according to the guidelines laid down by the MREC, Ministry of Health, Malaysia. Permission to use the secondary data was approved by the State Health Department of Negeri Sembilan.

## Results

### Baseline characteristics of patients

Among the 17,592 patients, 51.4% of them were younger adults below 60 years with a mean age of 59.1 ± 10.6 years. There were more females (56.3%), Malays (64.9%), non-smokers (93.8%), and 46.4% of the patients had diabetes for less than 5 years (Table 1). Sixty-eight point seven percent were overweight and obese, 83.0% had hypertension, and 78.4% had dyslipidaemia. Diabetes complications occurred in 0.9–5.4% of patients. Oral hypoglycaemic agent (OHA) alone was the primary diabetes treatment modality (70.0%), and 80.2% of the patients were treated with antihypertensive agents. Seventy-two percent of them were on lipid-lowering agents, while 28.9% were prescribed with antiplatelet agents. Altogether, 43.6% of the patients had polypharmacy.

**Table 1:** Baseline characteristics of patients, n = 17,592.

Characteristics	n (%)
**Age (years)**	
Mean ± standard deviation	59.1 ± 10.6
18–49	3016 (17.1)
50–59	6025 (34.3)
≥ 60	8551 (48.6)
**Sex**	
Male	7690 (43.7)
Female	9902 (56.3)
**Ethnicity**	
Malay	11,413 (64.9)
Chinese	2615 (14.9)
Indian	3449 (19.6)
Others	115 (0.6)
**Smoking**	
Smoker	1098 (6.2)
Non-smoker	16,495 (93.8)
**Duration of diabetes (years)**	
Median (inter-quartile range)	5.0 (7.0)
< 5	8162 (46.4)
5–10	6298 (35.8)
> 10	3132 (17.8)
**BMI category (n = 16,834)**	
Mean ± standard deviation (kg/m^2^)	28.0 ± 5.1
Normal (18.5–24.9 kg/m^2^)	4557 (25.9)
Underweight (< 18.5 kg/m^2^)	196 (1.1)
Overweight (25.0–29.9 kg/m^2^)	6810 (38.7)
Obese (≥ 30.0 kg/m^2^)	5271 (30.0)
**Hypertension**	
Yes	14,599 (83.0)
**Dyslipidaemia**	
Yes	13,792 (78.4)
**Nephropathy**	
Yes	944 (5.4)
**Retinopathy**	
Yes	482 (2.7)
**Foot complication**	
Yes	161 (0.9)
**Diabetes treatment modality**	
Lifestyle modification only	445 (2.5)
Oral hypoglycaemic agent (OHA) only	12,306 (70.0)
Insulin only	1041 (5.9)
Both OHA and insulin	3800 (21.6)
**Antihypertensive agents**	
Yes	14,109 (80.2)
**Lipid-lowering agents**	
Yes	12,688 (72.1)
**Antiplatelet agents**	
Yes	5092 (28.9)
**Polypharmacy**	
Yes	7673 (43.6)

### Descriptive statistics

The A1C values were unconditional and without regard to time (Table 2). The changing number of patients’ observations in each year reflected the dynamic membership of an open cohort and the random sampling of clinical audits.

**Table 2:** Mean A1C and proportion of patients achieving A1C goals, total n = 17,592.

	2013 n = 8247	2014 n = 9409	2015 n = 9950	2016 n = 10,656	2017 n = 10,219
Mean ± SD (%)	8.03 ± 2.06	7.94 ± 2.03	7.95 ± 2.11	7.90 ± 2.12	8.04 ± 2.13
Proportion achieving A1C < 7.0%, % with 95% CI	38.6% 37.6–39.7	40.4% 39.4–41.4	40.7% 39.8–41.7	42.6% 41.6–43.5	39.4% 38.5–40.4

### Five-year trends of A1C

**Figure 1:**
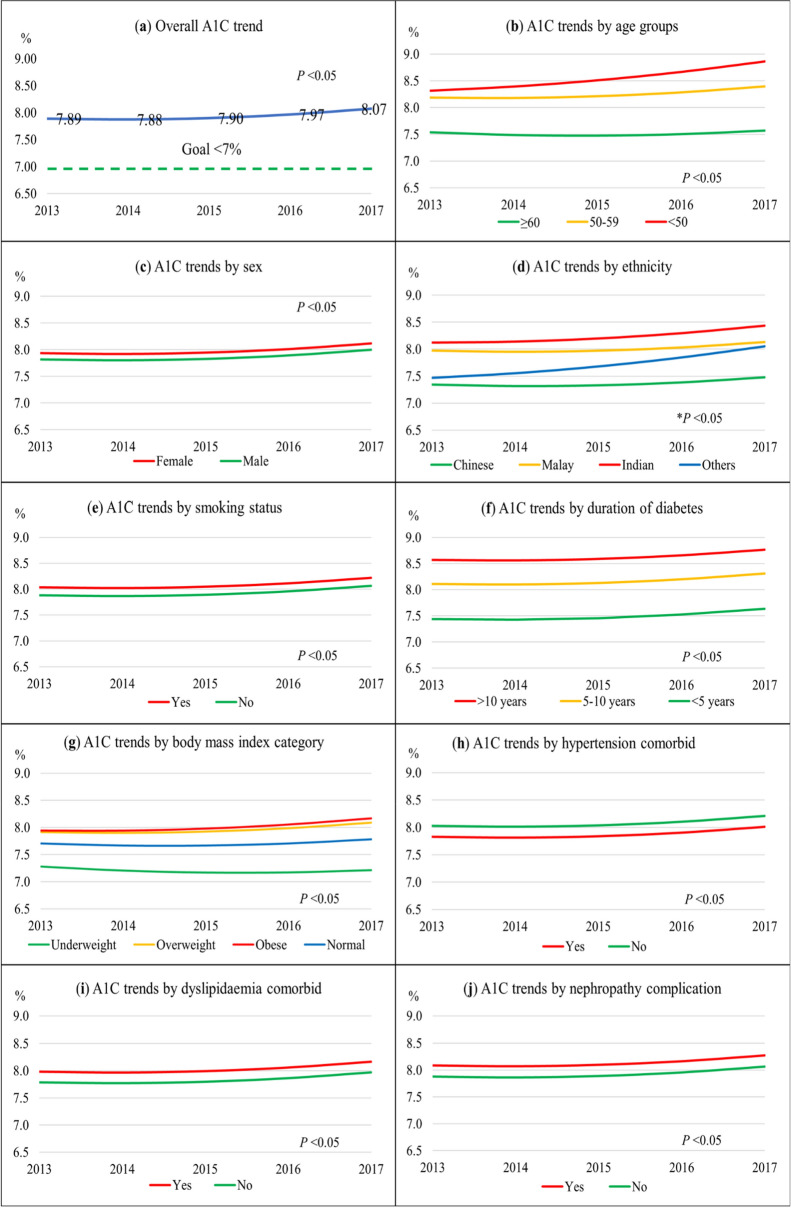

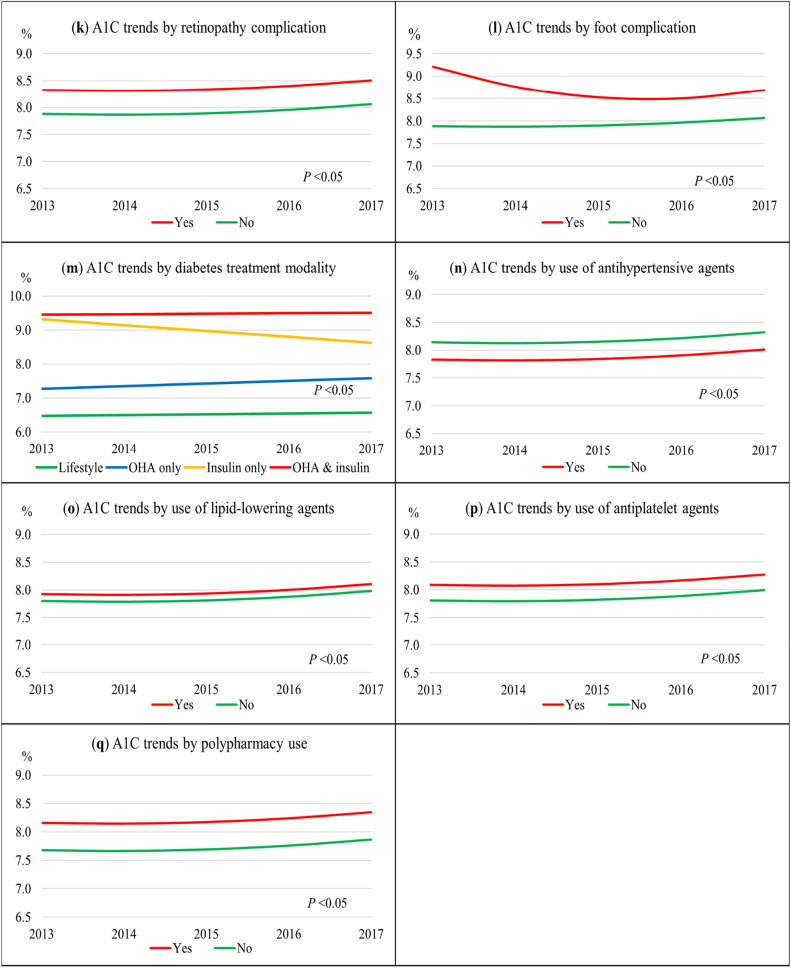
Overall A1C trend from 2013 to 2017 and A1C trends stratified by predictors. (**a**) Overall A1C trend; (**b**) By age groups; (**c**) By sex; (**d**) By ethnicity; (**e**) By smoking status; (**f**) By diabetes duration; (**g**) By BMI categories; (**h**) By hypertension; (**i**) By dyslipidaemia; (**j**) By diabetes nephropathy; (**k**) By diabetes retinopathy; (**l**) By diabetes foot complication; (**m**) By diabetes treatment modality; (**n**) By antihypertensive agents use; (**o**) By lipid-lowering agents use; (**p**) By antiplatelet agents use; (**q**) By polypharmacy use. Growth curves in all panels were statistically significant for trends and predictors. *Statistically significant for all except for ‘other ethnicities’.

Figure 1 depicts the univariate linear mixed-effect models for A1C trends, while Supplementary Table S1 details the statistical results. The significant linear effect for A1C trend was negative (parameter estimate = − 0.04%, *P* = 0.021) while the significant quadratic effect was positive (parameter estimate = 0.02%, *P* < 0.001). The resultant U-shaped curve showed an overall absolute A1C increase of 0.18%. (Fig. 1a) The gap between the A1C trend and treatment goal represented the shortfalls from the A1C goal.

All 16 predictors showed statistically significant differences between groups (Fig. 1b–q). Younger age groups, females, Malay and Indian ethnicities, smokers, longer duration of diabetes, higher BMI categories, patients with dyslipidaemia, nephropathy, retinopathy, and foot complication, and patients treated with insulin, lipid-lowering agents, antiplatelet agents, and polypharmacy had higher A1C trends than their respective counterparts. On the other hand, T2D patients with hypertension and those on antihypertensive agents had lower A1C trends.

The biggest initial differences in A1C between groups were for these variables: duration of diabetes, foot complication, and diabetes treatment modality. Patients with diabetes duration for > 10 years had higher A1C by 1.1% than patients with < 5 years of diagnosis (Fig. 1f). Although patients with foot complications had a large A1C difference initially (1.3%), the gap narrowed over time to < 1% (Fig. 1l). Compared to patients on OHA treatment alone, patients treated with insulin only, and both OHA and insulin had higher baseline A1C by 2.0% and 2.2%, respectively (Fig. 1m).

Age groups, ethnicity, BMI categories, foot complication, and diabetes treatment modality had significant interactions with time in predicting the A1C trends. While older adults exhibited a relatively flat curve, the younger age groups showed a steeper increase in A1C, especially in patients below 50 years whose A1C trend almost reached 9%, 18–49 years × time, *P* < 0.001 (Fig. 1b). Indian ethnic patients had the poorest A1C trend, followed by Malays, other ethnicities, and Chinese patients. Indian patients also had a significantly greater A1C growth rate than Chinese patients, Indian × time, *P* = 0.012 (Fig. 1d). For the BMI category, underweight patients showed an overall reduction of A1C trend. In contrast, obese patients had the highest A1C trend and a significant positive growth rate of A1C, obesity × time, *P* < 0.001 (Fig. 1g). Compared to patients on OHA alone whose A1C trend increased by 0.31% from 7.27 to 7.58%, patients treated with insulin alone had a considerable linear reduction of A1C by 0.69% from 9.32 to 8.63% (Fig. 1m). Patients treated with both OHA and insulin had a relatively flat curve as the A1C trend remained elevated near 9.5%.

### Predictors for A1C trend

**Figure 2:**
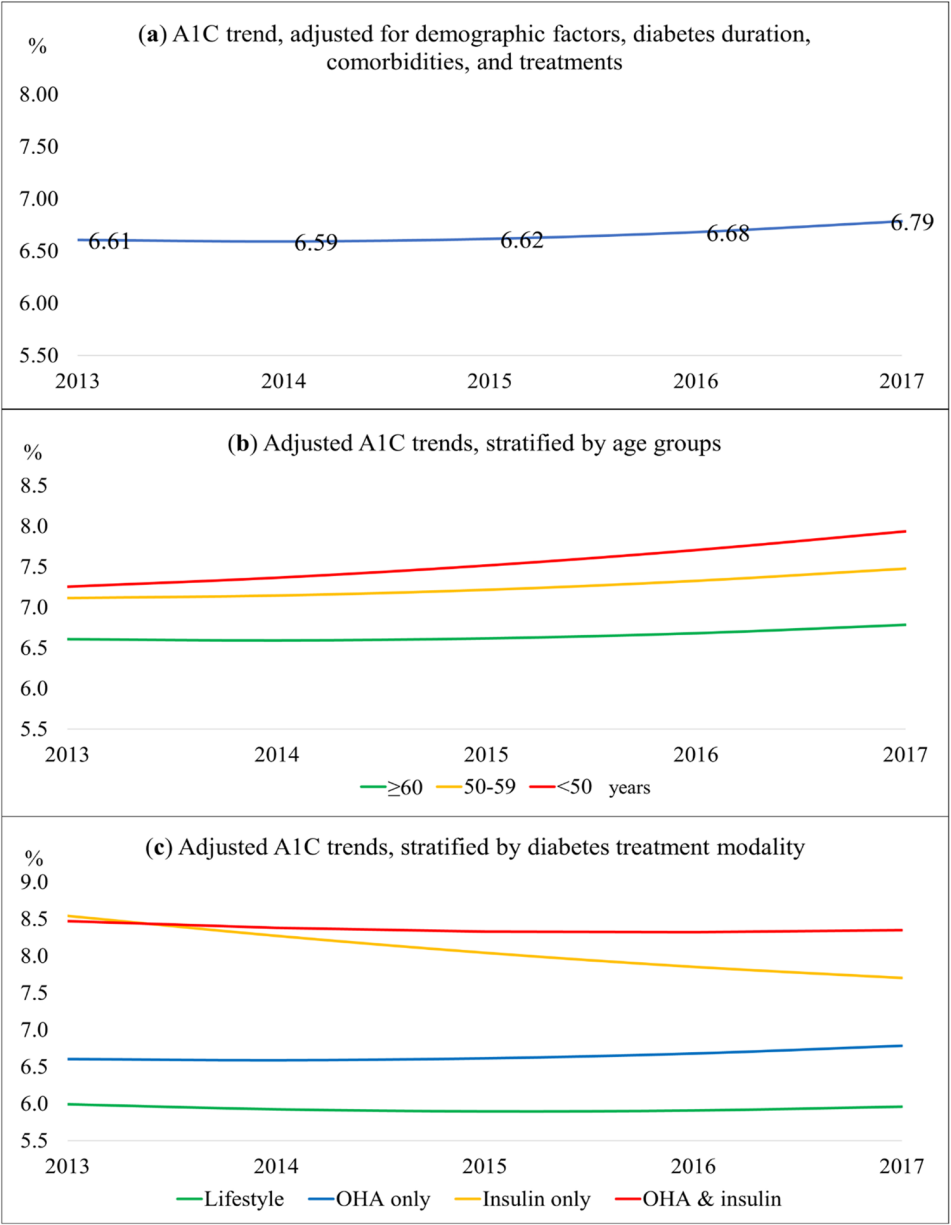
Multivariate linear mixed-effects model for A1C trends. (**a**) The adjusted baseline model was for Chinese ethnicity, aged ≥ 60, diabetes duration for < 5 years, normal BMI category, without hypertension, treated with OHA alone, not on antihypertensive agents, and no polypharmacy. (**b**) Adjusted A1C trends, stratified by age groups. (**c**) Adjusted A1C trends, stratified by diabetes treatment modality.

In the multivariate model, growth parameters were significant at the intercept, linear, and quadratic time trajectories (Table 3 and Fig. 2). The U-shaped curve showed an absolute net A1C increase of 0.18% over 5 years (Fig. 2a).

**Table 3:** Predictors for A1C trend from 2013 to 2017.

Fixed effects	A1C (%)	95% CI (%)	*P*-value
Intercept	6.61	6.51–6.70	< 0.001
Time	− 0.03	− 0.07 to − 0.002	0.040
Time^2^	0.02	0.01–0.03	< 0.001
**Ethnic**			
Malay	0.46	0.40–0.53	< 0.001
Chinese	0		
Indian	0.50	0.42–0.58	< 0.001
Others	− 0.02	− 0.34 to 0.31	0.920
**Disease duration (years)**			
< 5	0		
5–10	0.41	0.35–0.46	< 0.001
> 10	0.62	0.54–0.69	< 0.001
**Body mass index category**			
Underweight	− 0.38	− 0.60 to − 0.16	0.001
Normal	0		
Overweight	0.06	− 0.002 to 0.11	0.058
Obese	0.01	− 0.05 to 0.08	0.679
**Hypertension: yes**	− 0.14	− 0.20 to − 0.07	< 0.001
**Use of antihypertensive agent: yes**	− 0.24	− 0.31 to − 0.16	< 0.001
**Polypharmacy: yes**	0.25	0.19–0.30	< 0.001
**Age groups (years)**			
18–49	0.65	0.56–0.74	< 0.001
50–59	0.51	0.44–0.58	< 0.001
≥ 60	0		
**Age group × time**			
18–49 × time	0.13	0.10–0.15	< 0.001
50–59 × time	0.05	0.02–0.07	< 0.001
≥ 60 × time	0		
**Diabetes treatment modality**			
Lifestyle modification only	− 0.61	− 0.81 to − 0.42	< 0.001
OHA only	0		
Insulin only	1.94	1.81–2.07	< 0.001
OHA and insulin	1.86	1.79–1.94	< 0.001
**Diabetes treatment modality × time**			
Lifestyle modification only × time	− 0.05	− 0.12 to 0.01	0.114
OHA only × time	0		
Insulin only × time	− 0.26	− 0.30 to − 0.21	< 0.001
OHA and insulin × time	− 0.07	− 0.10 to − 0.05	< 0.001

Unstructured error covariance structure was the best structure that fitted the data. The model fits were 174,700.4 for − 2 log likelihood, 174,778.4 for Akaike’s Information Criterion (AIC), and 175,119.6 for Schwarz’s Bayesian Criterion (BIC).

Among the three main ethnicities, Indian ethnic patients had the highest baseline A1C, followed by Malay and Chinese patients. Duration of diabetes was an independent predictor at the intercept whereby longer diabetes duration was associated with increasing mean A1C. Age had a weak correlation with the duration of diabetes, Spearman’s correlation coefficient, *R*_*s*_ = 0.214, *P* < 0.001. Older patients might not necessarily have longer diabetes duration because of different age of diabetes onset. Compared to normal BMI category, underweight patients had lower baseline A1C. Although both overweight and obese patients had higher A1C values, the results were not statistically significant. Patients with hypertension and those treated with antihypertensive agents were independently associated with lower baseline A1C. On the other hand, patients with polypharmacy had higher baseline A1C.

Age groups and diabetes treatment modality were independent predictors for a linear change in the A1C trend. In 2013, compared to older adults, patients below 50 and 50–59 years had higher A1C by 0.65% and 0.51%, respectively. The gaps between age groups significantly widened over time (Fig. 2b). While the older adults showed a relatively flat curve, younger age groups had a steeper A1C increase, especially those in the below 50 years category.

Compared to patients treated with OHA only, patients on insulin alone and those with both OHA and insulin had higher baseline A1C by 1.94% and 1.86%, respectively (Fig. 2c). Patients on OHA alone had an overall A1C increase of 0.18% from 6.69 to 6.87%. In contrast, patients treated with insulin alone had a considerable linear reduction of A1C by 0.84% from 8.62 to 7.78%. The A1C trend in patients on both OHA and insulin reduced by 0.12% from 8.55 to 8.43%.

### Post hoc analysis

In this study, Chinese ethnic patients had the best A1C control. They were substantially older with 65.9% aged ≥ 60 years. Their mean age of 63.5 ± 10.7 years was almost 5 and 7 years higher than Malay and Indian patients, respectively (Supplementary Table S2). There were more males (52.5%) among Chinese patients, and they had the lowest mean BMI (26.5 ± 4.5 kg/m^2^). Chinese patients had the highest proportion of normal weight (37.7%) and the lowest obesity percentage (19.8%). The majority of them had hypertension (87.8%) and their usage of antihypertensive agents (84.6%) was the highest. Antiplatelet use (31.9%) and polypharmacy (45.2%) were more common among them. Moreover, Chinese patients had the lowest proportion of loss to follow-up (6.2%).

Among the three major ethnicities, the Malay ethnic patients had the highest mean body mass index (28.5 ± 5.2 kg/m^2^) and obesity percentage (34.9%). The majority of them had dyslipidaemia (79.6%) and were treated with lipid-lowering agents (73.4%). Malay patients had the highest loss of follow-up (8.3%). On the other hand, the Indian ethnic patients were the youngest at the mean age of 56.9 ± 10.2 years; 23.2% of them were below 50 years old. However, they had the longest duration of diabetes (median of 6 years with an interquartile range of 7 years), reflecting the earliest diabetes onset among the three ethnicities. The overall use of insulin was highest among Indian patients (29.9%). Despite the different characteristics between the ethnicities, there were no significant differences in diabetes complications.

Patients aged below 50 years old had the poorest A1C control. Between the three age categories, there were higher proportions of females (58.8%), Indians (26.5%), smokers (7.2%), and diabetes duration below 5 years (61.5%) in those aged below 50 years (Supplementary Table S3). The mean BMI was the highest among patients below 50 years category at 29.7 ± 5.6 kg/m^2^, of whom 37.2% were overweight and 43.8% obese. Patients below 50 years had the lowest proportions of hypertension (64.8%), dyslipidaemia (69.1%), diabetes nephropathy (2.9%), and retinopathy (1.6%). The use of antihypertensive (61.7%), lipid-lowering (63.1%), antiplatelet agents (18.3%), and polypharmacy (27.8%) was also the lowest among them. As comparison, older adults ≥ 60 years had the highest proportions of hypertension (90.6%), dyslipidaemia (81.5%), nephropathy (7.2%), retinopathy (3.6%), use of antihypertensive (87.8%), lipid-lowering (75.1%), antiplatelet agents (33.8%), and polypharmacy (49.4%). However, the overall use of insulin treatment was highest (31.1%) among patients below 50 years, followed by 50–59 years (30.1%) and ≥ 60 years categories (24.4%). Patients below 50 years category also had the highest proportion of loss to follow-up (9.4%).

## Discussion

The characteristics of our patients closely resemble those in the National Diabetes Registry, with more females, Malays, non-smokers, patients with comorbid hypertension, and patients with dyslipidaemia^12^. The mean age and the median diabetes duration were also similar to patients in the registry^12^.

The important observation from this study was that the mean A1C had changed from 8.3% in 2009 to 8.1% in 2017^12,13^. This trend indicated little improvement in the quality of diabetes care in Malaysia over the years. Nevertheless, the A1C trend was consistently above 8%. This is generally considered unacceptable by all clinical guidelines reviewed by the International Diabetes Federation^17^.

Potential factors that may hinder glycaemic improvement include time and resource constraints faced by the doctors during the consultation, similarly as reported in Singapore^10^. In 2019, after the Auditor-General’s report highlighted issues such as lack of facilities, a low budget, and personnel shortage in Malaysia’s Ministry of Health, the Director-General of Health acknowledged that the ministry was “underfunded, understaffed, underpaid, overworked, overstretched and with facilities overcrowded with patients”^25^. Patients’ health literacy on diabetes treatment goals may partly explain our suboptimal A1C trend, as reported in Singapore^10^. A local study found that only 3.6% of T2D patients had good knowledge of diabetes^26^.

Similar to our study, the overall glycaemic trend has deteriorated in the USA^27^. One postulated reason was the change in diabetes treatment guidelines in 2009; the A1C goal shifted from intensive glycaemic control for all patients to individualisation of glycaemic goals according to age and multimorbidity^28^. The same reason may partly explain the U-shaped curve and overall declining performance among our patients. The Malaysia’s previous clinical guideline set a sole treatment goal of < 6.5% for all patients^29^, and this was superseded by individualised A1C goals in the 2015 guideline^30^. The A1C trend increased from 2015 onwards because the treatment targets were less stringent for selected groups of T2D patients. For example, an A1C target of 7.1–8.0% is set for patients at higher-risk of developing hypoglycaemia, those with limited life expectancy, and those with advanced complications or extensive comorbid conditions^30^.

A repeated cross-sectional analysis over 7 years in Catalonia, Spain, also reported an overall increase in the A1C trend^31^. Clinical inertia—a delay in treatment intensification among patients with poor glycaemic control—was postulated as the primary reason for the A1C increase among their patients^31^. Clinical inertia may also play a role in our cohort as a recent study reported evidence of clinical inertia in the management of T2D patients in Malaysia^32^.

Each 1% reduction in A1C is associated with a 21% lower risk of any diabetes-related endpoints such as diabetes-related death, myocardial infarction, stroke, and microvascular complications^6^. Hence, the 0.18% increase in A1C in our patients over the study period may translate to 3.8% higher risks of diabetes complications, including death. Assuming a standard and common A1C goal of 7% for all patients, the excess mean A1C of 1.07% in 2017 represented 22.5% higher risks of diabetes-related endpoints in our patients^6,17^. These morbidity and mortality outcomes may be preventable if additional attention is given to control their glycaemia^9^

In this study, Chinese ethnic patients had the overall best A1C control compared to Malays and Indians, as reported in a previous study^33^. Post hoc analysis showed that Chinese ethnic patients had the lowest percentage of loss to follow-up, which may imply better health behaviour. They also had the lowest mean BMI, which may partly explain the better glycaemic control. A study reported that Chinese ethnic patients had the highest proportion of adequate health literacy and were 4.4 times more likely to have adequate health literacy levels than Malay ethnic patients^34^. Besides that, higher median household income (approximately 25%) among the Chinese population compared to the national average may partially explain the ethnic differences in our study^16^.

Meanwhile, among the three main ethnic groups, our Indian ethnic patients developed T2D at younger ages and had the longest diabetes duration; these may partly account for their poorest A1C control. Asian Indians have higher insulin resistance, and the age-related decline in pancreatic beta-cell function may become unmasked at an earlier age compared to Chinese and Malay ethnicities^35^. Furthermore, Indian T2D patients had the lowest literacy and knowledge scores compared to Chinese and Malay patients^26^.

Five novel subgroups of adult-onset diabetes with differing disease progression were identified using data-driven cluster analysis of six variables, including glutamate decarboxylase antibodies and homeostatic model assessment (HOMA) 2 estimates of beta-cell function and insulin resistance^36^. Could such clusters also exist among our multi-ethnic T2D patients? Our Chinese ethnic patients were substantially older and had the lowest A1C trend; this may coincide with the mild age-related diabetes cluster, which was the oldest and had the overall lowest A1C^36^. Our Malay ethnic patients with average A1C trend but highest BMI may correspond to the obesity-related diabetes cluster, which was characterised by obesity and moderate A1C progression^36^. Our Indian ethnic patients with the highest A1C trend were the youngest and had the highest proportion of insulin use. They may match the severe autoimmune diabetes or severe insulin-deficient diabetes clusters that had young ages at onset and highest A1C progression, or the severe insulin-resistant diabetes cluster with high insulin resistance^36^.

As each cluster differs in disease progression, risks of diabetes complications, and treatment response, the sub-stratification suggests that precision medicine can be used in diabetes management to tailor drug choice and target early treatment to patients^36,37^. Nevertheless, while the new clusters help in personalising management, it may not be cost-effective and practical to conduct widespread testing of antibodies and C-peptides in a country with a high diabetes burden^38^. Prediction models based on simple clinical features helped select therapy for individual patients, predict specific outcomes, and are more useful clinically to stratify patients than data-driven clusters^37^. We recommend similar studies using simple clinical measures in multi-ethnic populations to allow precise management of T2D patients. We acknowledge that differences in socioeconomic status, access to healthcare, genetics, sociocultural and psycho-behavioural factors are other potential explanations for the ethnic disparities^33^. However, these factors were not captured by the registry, thus making this a major limitation of the present study.

Our youngest patients had the poorest A1C, as reported in other studies^39–41^. However, unlike other studies, our study also factored in the growth rates where the youngest patients exhibited the highest A1C growth rate while older adults showed a relatively flat curve. Early tight glycaemic control reduces diabetes microvascular and macrovascular complications in the long-term; nevertheless, our younger patients who had the most to gain may not benefit from the legacy effect^7^. The deleterious effect of poor A1C control occurs across generations; suboptimal glycaemia during pregnancy is associated with adverse maternal and foetal outcomes^42^.

There are many potential factors for lower performance among younger patients, especially those aged 18–49 years. A study reported that younger patients had lesser in-person contact time with their healthcare providers and lower adherence to metformin^43^. They may experience distinct psychosocial barriers such as difficulty to cope with T2D diagnosis, stress, depression, poorer diet, lower diabetes self-efficacy, preference for ‘natural approaches’ as treatment, and resistance to medication initiation^43^. In addition, low motivation and time constraints due to job commitments may reduce their adherence to a healthy lifestyle, medication, and clinic visits^44^.

The traditional health delivery service might not be appropriately designed to meet the needs and preferences of younger patients such as online patient portals^43^. Most of the public health clinics in Malaysia only operate during office hours and are less accessible to younger patients who are more likely to be formally employed^45^. Our patients aged 18–49 years had the highest follow-up non-adherence. Hence, telemedicine using telephone, video, or email can facilitate provider-patient interaction at a time that fit young patients’ schedules^43^. Digital health applications on nutrition, physical activity, and glucose monitoring can be used to increase diabetes self-management among younger adults^46^.

Furthermore, the inherent differences in the pathophysiology of T2D at different ages of onset can partly explain the difference in glycaemic control. Young age at diabetes onset is associated with more aggressive diabetes^47^ and may be associated with severe autoimmune diabetes and severe insulin-deficient diabetes clusters as described above^36^. The higher proportion of insulin use among our younger patients may reflect more severe disease.

The cohort effect—the variation in the risk of a health outcome according to the year of birth—may partly explain the worst A1C control in our youngest patients^48^. The accelerated phase of industrialisation and urbanisation in recent decades has changed the dietary habits and sedentary lifestyles of people^49^. Exposure to poorer food consumption patterns and lower physical activity levels since young may partially explain the poorer A1C trend among our younger patients, who had the highest proportion of obesity^49^.

Patients with longer duration of diabetes had poorer A1C as consistently reported in other studies^39–41^. Duration of diabetes reflects the natural progression of diabetes severity as T2D is a chronic disease with increasing cellular insulin resistance and non-functioning pancreatic beta cells over time^50^. Although statistically insignificant in our adjusted model, overweight and obese patients had higher A1C, as reported elsewhere^39,41^. In T2D, the relationship between A1C and BMI was mostly due to BMI influencing glycaemia through insulin resistance or secretion^51^. Hence, the statistical insignificance of overweight and obese BMI is most likely because of the adjustment by insulin therapy in our multivariate model. Hypertensive patients and those treated with antihypertensive agents had lower adjusted A1C, which could be due to more aggressive treatment plans in patients with comorbidities^30^.

Diabetes treatment modality was a significant predictor of linear growth parameters. Patients treated with insulin alone, and both OHA and insulin had a drop in the A1C trend, whereas the A1C trend increased in patients on OHA alone, as reported in Iran^52^. However, despite the A1C changes, the discrepancy between treatment modality persisted. The counter-intuitive findings where more intensive treatments (insulin and polypharmacy use) were associated with higher A1C curves may be explained by these factors: (a) confounding by severity, where patients with more severe disease are likely to receive more intensive treatments, and when comparing with interventions, the more intensive intervention will appear to result in poorer outcomes^53^; (b) inadequate adjustment of the regression models as some confounders were not captured and could not be included in the model^40^; (c) the patient’s profile, such as age, sex, comorbidity, and disease severity, influences treatment effectiveness^54^; (d) the use of multiple medications can lead to inappropriate drug use, under prescription, low adherence, and side effects^55,56^; (e) poor medication adherence is a reason for the disconnect between drug efficacy in clinical trials and drug effectiveness in the real world^27^; and (f) clinical inertia—insulin is given too late after chronic exposure to hyperglycaemia, thus marking patients with poor A1C control. The poor outcome reflects the fact that insulin therapy is initiated too late rather than its appropriate use^57^.

This study has several limitations. First, the effect of self-management such as healthy eating, active living, medication adherence, self-monitoring of blood glucose, and diabetes self-management education, on A1C control was not explored as these factors were not captured by the National Diabetes Registry. Other information on treatment regimens such as individual drug types and dosages were also not available; hence changes in the treatment regimen could not be factored into the multivariate model. Second, we did not account for individualised A1C based on patients’ characteristics. Third, while the secondary data reflects the real-world clinical practice, measurement errors could present because of the absence of standardisation procedures.

To the very best of our knowledge, this study is the first large population-based longitudinal study in Malaysia to determine the predictors of A1C trend among T2D patients. We identified the high-risk populations by demographic characteristics; younger patients and those of Indian and Malay ethnicity had poorer A1C trends. In precision public health, it is critical to identify precisely the subpopulations to customise interventions to improve the overall population health^14^. We believe our study findings will add to the pool of local evidence to inform evidence-based policymaking and the development of clinical guidelines in Malaysia. Population health management can improve diabetes care outcomes and potentially ameliorate ethnic disparities in health care; the approach proactively identifies at-risk patients through disease registries and electronic health record data for interventions to be given^58^. The existing National Diabetes Registry may be leveraged to serve similar purposes.

From 2013 to 2017, the A1C trend has increased marginally in a cohort of T2D patients in Malaysia. The gap between real-world A1C performance and treatment goal represents a potentially avoidable burden of CVD, which can be improved. The predictors for higher baseline A1C are non-Chinese ethnicity, younger age groups, longer diabetes duration, treatment with insulin, polypharmacy use, without hypertension and not on antihypertensive agents. Younger age groups predicted a linear increase in the A1C trend, whereas patients on insulin treatment predicted a linear decrease in the A1C trend. In conclusion, our study showed that younger adults and non-Chinese patients are high-risk populations and should be targeted for interventions.

## Supplementary Information

Supplementary Tables.

## Data Availability

The National Diabetes Registry dataset retrieved and analysed in this study is not available publicly due to local ethics regulation and could be obtained via written permissions to the Director General of Health, Malaysia.

## Abbreviations

A1C: Glycosylated haemoglobin A1c
AIC: Akaike’s information criterion
ANOVA: Analysis of variance
BIC: Bayesian information criterion
BMI: Body mass index
CVD: Cardiovascular disease
GLM: General linear model
HOMA: Homeostatic model assessment
IQR: Inter-quartile range
LMM: Linear mixed-effects model
MCAR: Missing completely at random
MREC: Medical Review and Ethics Committee
NMRR: National Medical Research Register
OHA: Oral hypoglycaemic agent
SD: Standard deviation
SPSS: Statistical Package for the Social Sciences
T2D: Type 2 diabetes
UKPDS: United Kingdom Prospective Diabetes Study
