# Supplementary material for: Predictors of glycosylated haemoglobin A1C trend among type 2 diabetes patients in a multi-ethnic country
Source: Sci Rep. 2021 Mar 24;11:6803. doi: 10.1038/s41598-021-86277-0 (PMC7991644; doi:10.1038/s41598-021-86277-0)
Supplement: Supplementary file 1 — Supplementary Tables. [file 41598_2021_86277_MOESM1_ESM.docx]

**Predictors of glycosylated haemoglobin A1C trend among type 2 diabetes patients in a multi-ethnic country**

Kim Sui Wan^1^, Noran Naqiah Hairi^1*^, Feisul Idzwan Mustapha^2^, Khalijah Mohd Yusof^3^, Zainudin Mohd Ali^4^, Foong Ming Moy^1^

^1^Centre for Epidemiology and Evidence-Based Practice, Department of Social and Preventive Medicine, Faculty of Medicine, University of Malaya, Malaysia

^2^Disease Control Division, Ministry of Health, Malaysia

^3^State Health Department of Johor, Malaysia

^4^State Health Department of Negeri Sembilan, Malaysia

*Corresponding author: noran@um.edu.my

**Supplementary Table S1: Univariate linear mixed-effect models for A1C trends, n = 17,592**

|  | Fixed effects | A1C estimates | 95% CI | *P*-value |
| --- | --- | --- | --- | --- |
| (a) Overall trend | Intercept  Time  Time^2^ | 7.89  -0.04  0.02 | 7.85 – 7.93  -0.66 – -0.05  0.01 – 0.03 | <0.001  0.021  <0.001 |
| (b) Age groups, years | Intercept  Time  Time^2^  18–49  50–59  ≥60  18–49*time  50–59*time  ≥60*time | 7.54  -0.07  0.02  0.78  0.65  0  0.13  0.04  0 | 7.49 – 7.59  -0.10 – -0.04  0.01 – 0.03  0.68 – 0.87  0.58 – 0.72  0.10 – 0.16  0.02 – 0.07 | <0.001  <0.001  <0.001  <0.001  <0.001  <0.001  <0.001 |
| (c) Sex | Intercept  Time  Time^2^  Female  Male | 7.82  -0.04  0.02  0.12  0 | 7.77 – 7.87  -0.07 – -0.01  0.01 – 0.03  0.07 – 0.18 | <0.001  0.022  <0.001  <0.001 |
| (d) Ethnic | Intercept  Time  Time^2^  Malay  Indian  Others  Chinese  Malay*time  Indian*time  Others*time  Chinese*time | 7.34  -0.05  0.02  0.63  0.78  0.13  0  0.01  0.04  0.11  0 | 7.26 – 7.427  -0.09 – -0.01  0.01 – 0.03  0.54 – 0.72  0.66 – 0.89  -0.33 – 0.59  -0.02 – 0.03  0.01 – 0.08  -0.03 – 0.25 | <0.001  0.015  <0.001  <0.001  <0.001  0.587  0.667  0.012  0.111 |
| (e) Smoker | Intercept  Time  Time^2^  Yes  No | 7.88  -0.04  0.02  0.15  0 | 7.85 – 7.92  -0.07 – -0.01  0.01 – 0.03  0.03 – 0.27 | <0.001  0.022  <0.001  0.012 |
| (f) Duration of diabetes, years | Intercept  Time  Time^2^  <5  5–10  >10 | 7.44  -0.03  0.02  0  0.68  1.13 | 7.39 – 7.48  -0.06 – -0.0001  0.01 – 0.03  0.62 – 0.73  1.06 – 1.21 | <0.001  0.049  <0.001  <0.001  <0.001 |
| (g) Body mass index category | Intercept  Time  Time^2^  Underweight  Normal  Overweight  Obese  Underweight*time  Normal*time  Overweight*time  Obese*time | 7.77  -0.04  0.02  -0.52  0  0.18  0.19  -0.05  0  0.004  0.03 | 7.70 – 7.83  -0.08 – -0.01  0.01 – 0.03  -0.84 – -0.20  0.10 – 0.27  0.10 – 0.28  -0.15 – 0.05  -0.02 – 0.03  0.01 – 0.06 | <0.001  0.019  <0.001  0.001  <0.001  <0.001  0.359  0.786  0.014 |
| (h) Hypertension | Intercept  Time  Time^2^  Yes  No | 8.03  -0.04  0.02  -0.20  0 | 7.97 – 8.08  -0.07 – -0.01  0.01 – 0.03  -0.26 – -0.14 | <0.001  0.019  <0.001  <0.001 |
| (i) Dyslipidaemia | Intercept  Time  Time^2^  Yes  No | 7.79  -0.03  0.02  0.19  0 | 7.74 – 7.84  -0.065 – -0.005  0.01 – 0.03  0.14 – 0.25 | <0.001  0.024  <0.001  <0.001 |
| (j) Nephropathy | Intercept  Time  Time^2^  Yes  No | 7.88  -0.04  0.02  0.21  0 | 7.84 – 7.92  -0.07 – -0.01  0.01 – 0.03  0.09 – 0.33 | <0.001  0.022  <0.001  0.001 |
| (k) Retinopathy | Intercept  Time  Time^2^  Yes  No | 7.88  -0.04  0.02  0.44  0 | 7.84 – 7.92  -0.07 – -0.01  0.01 – 0.03  0.28 – 0.61 | <0.001  0.022  <0.001  <0.001 |
| (l) Foot complication | Intercept  Time  Time^2^  Yes  No  Yes*time  No*time  Yes*time^2^  No*time^2^ | 7.88  -0.03  0.02  1.33  0  -0.52  0  0.09  0 | 7.84 – 7.92  -0.061 – -0.001  0.01 – 0.03  0.93 – 1.73  -0.85 – -0.20  0.01 – 0.16 | <0.001  0.044  <0.001  <0.001  0.002  0.026 |
| (m) Diabetes treatment modality | Intercept  Time  Lifestyle only  OHA only  Insulin only  OHA & insulin  Lifestyle only*time  OHA only*time  Insulin only*time  OHA & insulin*time | 7.27  0.08  -0.79  0  2.04  2.18  -0.06  0  -0.25  -0.07 | 7.24 – 7.31  0.07 – -0.09  -0.99 – -0.60  1.92 – 2.17  2.11 – 2.26  -0.12 – 0.01  -0.29 – -0.21  -0.09 – -0.04 | <0.001  <0.001  <0.001  <0.001  <0.001  0.086  <0.001  <0.001 |
| (n) Use of antihypertensive agent | Intercept  Time  Time^2^  Yes  No | 8.14  -0.04  0.02  -0.31  0 | 8.07 – 8.21  -0.07 – -0.01  0.01 – 0.03  -0.38 – -0.24 | <0.001  0.020  <0.001  <0.001 |
| (o) Use of lipid-lowering agent | Intercept  Time  Time^2^  Yes  No | 7.80  -0.04  0.02  0.13  0 | 7.74 – 7.86  -0.07 – -0.01  0.01 – 0.03  0.07 – 0.19 | <0.001  0.021  <0.001  <0.001 |
| (p) Use of antiplatelet agent | Intercept  Time  Time^2^  Yes  No | 7.81  -0.03  0.02  0.28  0 | 7.77 – 7.85  -0.065 – -0.004  0.01 – 0.03  0.22 – 0.34 | <0.001  0.025  <0.001  <0.001 |
| (q) Polypharmacy status | Intercept  Time  Time^2^  Yes  No | 7.68  -0.03  0.02  0.48  0 | 7.63 – 7.72  -0.063 – -0.003  0.01 – 0.03  0.43 – 0.54 | <0.001  0.032  <0.001  <0.001 |

# **Supplementary Table S2:** **Baseline characteristics of patients by ethnicity, n = 17,592**

|  | | Malays | | Chinese | Indians | Others | *P*-value |
| --- | --- | --- | --- | --- | --- | --- | --- |
|  |  | n (column %) | | n (column %) | n (column %) | n (column %) |  |
|  |  | 11,413 (100) | | 2,614 (100) | 3,448 (100) | 117 (100) |  |
| **Age,** mean ± SD | | 58.8 ± 10.4 | | 63.5 ± 10.7 | 56.9 ±10.2 | 51.9 ± 10.2 | <0.001 |
|  | 18–49 years | 1,908 (16.7) | | 263 (10.1) | 799 (23.2) | 48 (40.5) | <0.001 |
|  | 50–59 years | 4,101 (35.9) | | 628 (24.0) | 1,249 (36.2) | 46 (39.7) |  |
|  | ≥60 years | 5,404 (47.3) | | 1,723 (65.9) | 1,400 (40.6) | 23 (19.8) |  |
| **Sex** | |  | |  |  |  | <0.001 |
|  | Male | 4,754 (41.7) | | 1,373 (52.5) | 1,516 (44.0) | 47 (40.2) |  |
|  | Female | 6,658 (58.3) | | 1,242 (47.5) | 1,932 (56.0) | 70 (59.8) |  |
| **Smoker** | |  | |  |  |  | 0.001 |
|  | Yes | 706 (6.2) | | 178 (6.8) | 196 (5.7) | 17 (14.7) |  |
|  | No | 10,706 (93.8) | | 2,437 (93.2) | 3,252 (94.3) | 100 (85.3) |  |
| **Duration of diabetes,** median (IQR) | | 5.0 (6.0) | | 5.0 (7.0) | 6.0 (7.0) | 3.0 (4.0) | <0.001 |
|  | <5 years | 5,540 (48.5) | | 1,143 (43.7) | 1,401 (40.6) | 79 (67.3) | <0.001 |
|  | 5–10 years | 4,098 (35.9) | | 900 (34.4) | 1,269 (36.8) | 31 (26.7) |  |
|  | >10 years | 1,775 (15.6) | | 572 (21.9) | 778 (22.6) | 7 (6.0) |  |
| **Body mass index**, kg/m^2^, mean ± SD (n = 16,834) | | 28.5 ± 5.2 | | 26.5 ± 4.5 | 27.6 ± 5.1 | 28.8 ± 5.1 | <0.001 |
|  | Underweight | 109 (1.0) | | 42 (1.7) | 45 (1.3) | 0 (0.0) | <0.001 |
|  | Normal | 2,562 (23.6) | | 949 (37.7) | 1,014 (30.4) | 31 (28.4) |  |
|  | Overweight | 4,405 (40.5) | | 1,028 (40.8) | 1,338 (40.1) | 39 (35.8) |  |
|  | Obese | 3,794 (34.9) | | 499 (19.8) | 940 (28.2) | 39 (35.8) |  |
| **Hypertension** | |  | |  |  |  | <0.001 |
|  | Yes | 9,606 (84.2) | | 2,295 (87.8) | 2,608 (75.6) | 90 (76.7) |  |
|  | No | 1,806 (15.8) | | 319 (12.2) | 841 (24.4) | 27 (23.3) |  |
| **Dyslipidaemia** | |  | |  |  |  | <0.001 |
|  | Yes | 9,089 (79.6) | | 2,039 (78.0) | 2,577 (74.7) | 87 (74.4) |  |
|  | No | 2,323 (20.4) | | 576 (22.0) | 872 (25.3) | 30 (25.6) |  |
| **Nephropathy** | |  | |  |  |  | 0.516 |
|  | Yes | 630 (5.5) | | 140 (5.4) | 168 (4.9) | 7 (6.0) |  |
|  | No | 10,783 (94.5) | | 2,475 (94.6) | 3,281 (95.1) | 110 (94.0) |  |
| **Retinopathy** | |  | |  |  |  | 0.344 |
|  | Yes | 304 (2.7) | | 82 (3.1) | 95 (2.8) | 1 (0.9) |  |
|  | No | 11,108 (97.3) | | 2,533 (96.9) | 3,354 (97.2) | 116 (99.1) |  |
| **Foot complication** | | |  |  |  |  | 0.301 |
|  | Yes | | 112 (1.0) | 17 (0.7) | 32 (0.9) | 0 (0.0) |  |
|  | No | | 11,301 (99.0) | 2,598 (99.3) | 3,417 (99.1) | 117 (100.0) |  |
| **Diabetes treatment modality** | | |  |  |  |  | <0.001 |
|  | Lifestyle modification | | 295 (2.6) | 91 (3.5) | 55 (1.6) | 0 (0.0) |  |
|  | OHA only | | 7,945 (69.6) | 1,923 (73.5) | 2,362 (68.5) | 80 (68.4) |  |
|  | Insulin only | | 771 (6.8) | 128 (4.9) | 132 (3.8) | 11 (9.4) |  |
|  | Both OHA and insulin | | 2402 (21.0) | 473 (18.1) | 900 (26.1) | 26 (22.2) |  |
| **Antihypertensive agents** | |  | |  |  |  | <0.001 |
|  | Yes | 9,307 (81.5) | | 2,210 (84.6) | 2,503 (72.6) | 88 (75.9) |  |
|  | No | 2,106 (18.5) | | 404 (15.4) | 945 (27.4) | 28 (24.1) |  |
| **Lipid-lowering agents** | |  | |  |  |  | <0.001 |
|  | Yes | 8,375 (73.4) | | 1,868 (71.4) | 2,364 (68.6) | 81 (69.2) |  |
|  | No | 3,038 (26.6) | | 747 (28.6) | 1,084 (31.4) | 36 (30.8) |  |
| **Antiplatelet agents** | |  | |  |  |  | 0.005 |
|  | Yes | 3,236 (28.4) | | 833 (31.9) | 992 (28.8) | 31 (26.7) |  |
|  | No | 8,177 (71.6) | | 1,782 (68.1) | 2,456 (71.2) | 86 (73.3) |  |
| **Polypharmacy** | |  | |  |  |  | <0.001 |
|  | Yes | 5,062 (44.4) | | 1,181 (45.2) | 1,384 (40.1) | 47 (40.2) |  |
|  | No | 6,351 (55.6) | | 1,434 (54.8) | 2,065 (59.9) | 70 (59.8) |  |
| **Follow-up status** | |  | |  |  |  | <0.001 |
|  | Active follow-up | 10,307 (90.3) | | 2,435 (93.1) | 3,180 (92.2) | 99 (84.5) |  |
|  | Loss to follow-up | 951 (8.3) | | 161 (6.2) | 247 (7.2) | 14 (12.1) |  |
|  | All-cause mortality | 155 (1.4) | | 19 (0.7) | 21 (0.6) | 4 (3.4) |  |

# **Supplementary Table S3:** **Baseline characteristics of patients by age groups, n = 17,592**

|  | | 18–49 years | | 50–59 years | ≥60 years | *P*-value |
| --- | --- | --- | --- | --- | --- | --- |
|  |  | n (column %) | | n (column %) | n (column %) |  |
|  |  | 3,016 (100) | | 6,025 (100) | 8,551 (100) |  |
| **Age,** mean ± SD | | 43.1 ± 5.5 | | 55.0 ± 2.8 | 67.6 ± 6.3 | <0.001 |
| **Sex** | |  | |  |  | <0.001 |
|  | Male | 1,243 (41.2) | | 2,501 (41.5) | 3,946 (46.2) |  |
|  | Female | 1,773 (58.8) | | 3,524 (58.5) | 4,605 (53.8) |  |
| **Ethnicity** | |  | |  |  | <0.001 |
|  | Malay | 1,907 (63.2) | | 4,102 (68.1) | 5,405 (63.2) |  |
|  | Chinese | 263 (8.7) | | 628 (10.4) | 1,723 (20.2) |  |
|  | Indian | 799 (26.5) | | 1,249 (20.7) | 1,400 (16.4) |  |
|  | Others | 47 (1.6) | | 46 (0.8) | 23 (0.3) |  |
| **Smoker** | |  | |  |  | 0.001 |
|  | Yes | 217 (7.2) | | 404 (6.7) | 477 (5.6) |  |
|  | No | 2,799 (92.8) | | 5,621 (93.3) | 8,074 (94.4) |  |
| **Duration of diabetes,** median (IQR) | | 3.0 (5.0) | | 5.0 (6.0) | 6.0 (7.0) | <0.001 |
|  | <5 years | 1,853 (61.5) | | 2,946 (48.9) | 3,362 (39.3) | <0.001 |
|  | 5–10 years | 924 (30.6) | | 2,206 (36.6) | 3,169 (37.1) |  |
|  | >10 years | 239 (7.9) | | 873 (14.5) | 2,020 (23.6) |  |
| **Body mass index**, kg/m^2^, mean ± SD (n = 16,834) | | 29.7 ± 5.6 | | 28.7 ± 5.2 | 26.9 ± 4.6 | <0.001 |
|  | Underweight | 21 (0.7) | | 27 (0.5) | 148 (1.8) | <0.001 |
|  | Normal | 528 (18.2) | | 1,317 (22.7) | 2,711 (33.3) |  |
|  | Overweight | 1,079 (37.2) | | 2,370 (40.9) | 3,362 (41.3) |  |
|  | Obese | 1,269 (43.8) | | 2,081 (35.9) | 1,921 (23.6) |  |
| **Hypertension** | |  | |  |  | <0.001 |
|  | Yes | 1,955 (64.8) | | 4,901 (81.3) | 7,743 (90.6) |  |
|  | No | 1,061 (35.2) | | 1,124 (18.7) | 808 (9.4) |  |
| **Dyslipidaemia** | |  | |  |  | <0.001 |
|  | Yes | 2,083 (69.1) | | 4,744 (78.7) | 6,965 (81.5) |  |
|  | No | 933 (30.9) | | 1,281 (21.3) | 1,586 (18.5) |  |
| **Nephropathy** | |  | |  |  | <0.001 |
|  | Yes | 86 (2.9) | | 245 (4.1) | 614 (7.2) |  |
|  | No | 2,930 (97.1) | | 5,780 (95.9) | 7,937 (92.8) |  |
| **Retinopathy** | |  | |  |  | <0.001 |
|  | Yes | 48 (1.6) | | 129 (2.1) | 305 (3.6) |  |
|  | No | 2,968 (98.4) | | 5,896 (97.9) | 8,246 (96.4) |  |
| **Foot complication** | | |  |  |  | 0.305 |
|  | Yes | | 29 (1.0) | 46 (0.8) | 86 (1.0) |  |
|  | No | | 2,987 (99.0) | 5,979 (99.2) | 8,465 (99.0) |  |
| **Antihypertensive agents** | |  | |  |  | <0.001 |
|  | Yes | 1,860 (61.7) | | 4,738 (78.6) | 7,510 (87.8) |  |
|  | No | 1,156 (38.3) | | 1,287 (21.4) | 1,041 (12.2) |  |
| **Lipid-lowering agents** | |  | |  |  | <0.001 |
|  | Yes | 1,902 (63.1) | | 4,368 (72.5) | 6,418 (75.1) |  |
|  | No | 1,114 (36.9) | | 1,657 (27.5) | 2,133 (24.9) |  |
| **Antiplatelet agents** | |  | |  |  | <0.001 |
|  | Yes | 553 (18.3) | | 1,649 (27.4) | 2,890 (33.8) |  |
|  | No | 2,463 (81.7) | | 4,376 (72.6) | 5,661 (66.2) |  |
| **Polypharmacy** | |  | |  |  | <0.001 |
|  | Yes | 839 (27.8) | | 2,610 (43.3) | 4,225 (49.4) |  |
|  | No | 2,177 (72.2) | | 3,415 (56.7) | 4,326 (50.6) |  |
| **Diabetes treatment modality** | | |  |  |  | <0.001 |
|  | Lifestyle modification | | 60 (2.0) | 122 (2.0) | 260 (3.0) |  |
|  | OHA only | | 2,017 (66.9) | 4,088 (67.9) | 6,204 (72.6) |  |
|  | Insulin only | | 160 (5.3) | 291 (4.8) | 590 (6.9) |  |
|  | Both OHA and insulin | | 779 (25.8) | 1,524 (25.3) | 1,497 (17.5) |  |
| **Follow-up status** | |  | |  |  | <0.001 |
|  | Active follow-up | 2,730 (90.5) | | 5,561 (92.3) | 7,730 (90.4) |  |
|  | Loss to follow-up | 282 (9.4) | | 427 (7.1) | 664 (7.8) |  |
|  | All-cause mortality | 4 (0.1) | | 37 (0.6) | 157 (1.8) |  |
